# Whole Blood Storage in CPDA1 Blood Bags Alters Erythrocyte Membrane Proteome

**DOI:** 10.1155/2018/6375379

**Published:** 2018-11-08

**Authors:** Amna Mohamed Al-Thani, Sven Christian Voss, Afnan Saleh Al-Menhali, Andrei Barcaru, Peter Horvatovich, Hind Al Jaber, Zoran Nikolovski, Aishah Latiff, Costas Georgakopoulos, Zeyd Merenkov, Jordi Segura, Mohammed Alsayrafi, Morana Jaganjac

**Affiliations:** ^1^Anti-Doping Lab Qatar, Doha, Qatar; ^2^Department of Analytical Biochemistry, Research Institute of Pharmacy, University of Groningen, Antonius Deusinglaan 1, 9713 AV Groningen, Netherlands; ^3^Aspire Academy, Doha, Qatar; ^4^Blood Donation Unit, Hamad Medical Corporation, Doha, Qatar; ^5^IMIM-Hospital del Mar Research Institute, Barcelona, Spain

## Abstract

Autologous blood transfusion (ABT) has been frequently abused in endurance sport and is prohibited since the mid-1980s by the International Olympic Committee. Apart from any significant performance-enhancing effects, the ABT may pose a serious health issue due to aging erythrocyte-derived “red cell storage lesions.” The current study investigated the effect of blood storage in citrate phosphate dextrose adenine (CPDA1) on the red blood cell (RBC) membrane proteome. One unit of blood was collected in CPDA1 blood bags from 6 healthy female volunteers. RBC membrane protein samples were prepared on days 0, 14, and 35 of storage. Proteins were digested in gel and peptides separated by nanoliquid chromatography coupled to tandem mass spectrometry resulting in the confident identification of 33 proteins that quantitatively change during storage. Comparative proteomics suggested storage-induced translocation of cytoplasmic proteins to the membrane while redox proteomics analysis identified 14 proteins prone to storage-induced oxidation. The affected proteins are implicated in the RBC energy metabolism and membrane vesiculation and could contribute to the adverse posttransfusion outcomes. Spectrin alpha chain, band 3 protein, glyceraldehyde-3-phosphate dehydrogenase, and ankyrin-1 were the main proteins affected by storage. Although potential biomarkers of stored RBCs were identified, the stability and lifetime of these markers posttransfusion remain unknown. In summary, the study demonstrated the importance of studying storage-induced alterations in the erythrocyte membrane proteome and the need to understand the clearance kinetics of transfused erythrocytes and identified protein markers.

## 1. Introduction

Transfusion of whole blood or erythrocyte concentrates is considered blood doping and prohibited by the World Anti-Doping Agency (WADA) [1]. To detect doping with homologous blood, Anti-Doping Laboratories use flow cytometry to screen for erythrocyte surface markers which vary between individuals [2, 3]. In an attempt to detect doping by autologous blood transfusion (ABT), the Athlete Biological Passport approach is applied. Within this approach, hematological data of athletes are collected longitudinally and monitored over time, and deviations from the athletes' individual reference values can indirectly indicate usage of doping substance or method [4]. Unfortunately, due to limited sensitivity, ABT can still be abused by athletes to increase the oxygen delivery capacity to the tissue and subsequently enhance aerobic performance [5, 6]. Although there are performance-enhancing effects from applying the transfusion, one also needs to consider the possible adverse effects such as deep venous thrombosis and transfusion-related acute lung injury which might be connected to aging erythrocyte-derived “red cell storage lesions” [7]. Other side effects such as acute and delayed hemolytic reactions, blood-borne infections, or graft-versus-host disease are also related to incompatibilities with donor blood and can occur in cases of homologous blood transfusion [8].

Aging erythrocytes in stored blood undergo extensive remodeling of the membrane and marked structural changes. They display removal signals on their surface among which are neoantigens on band 3 protein and phosphatidylserine (PS). Both neoantigens on band 3 and PS when bound by autologous IgG or macrophage scavenger receptors, respectively, will trigger phagocytosis [9]. The oxidative injury of lipids and proteins that occurs with storage can lead to the formation of erythrocyte membrane microparticles and release of bioactive lipids from its membrane [10] and contribute to storage lesions [11].

Reactive oxygen species (ROS) are continuously generated in the vicinity of the ferrous hemoglobin molecule and released into the extracellular space. Erythrocyte superoxide dismutase and methemoglobin reductase can scavenge superoxide radicals and ferric methemoglobin contributing to the recovery from oxidative injury. However, stored erythrocytes have an impaired glycolytic pathway and decreased levels of glutathione—both needed to prevent the formation of extremely harmful hydroxyl radicals via the Fenton reaction [12]. Hydroxyl radicals derived by the Fenton reaction can mediate direct oxidation, in particular of sulfur-containing amino acids like methionine and cysteine [13], altering protein conformation and function. Protein carbonyls are other frequently used biomarkers of protein oxidation and oxidative stress as they are irreversible modifications [14]. One of the methods available for the assessment of carbonylated proteins is oxyblotting that gives information of the molecular weight of carbonylated proteins. Another approach, applied for this study, is to use the mass spectrometry technique that allows the identification of protein targets and sites of AA carbonyl modifications [15].

Different methodologies of blood storage are available. The most common way is to store the blood in a refrigerator, but RBCs can also be frozen when cryoprotected by glycerol. For refrigeration, blood can be stored as whole blood or separated into red blood cell concentrate and plasma with or without removal of leukocytes. Blood bags with different additives, like saline-adenine-glucose-mannitol (SAGM) or Erythro-Sol (E-Sol), affecting the storage time exist [16].

One possible method readily available to athletes, which does not require access to professional transfusion equipment, is the storage of whole blood in citrate phosphate dextrose adenine (CPDA1) blood bags at 4°C. However, blood stored in this way contains both platelets and leukocytes that can promote oxidative injury of erythrocytes during the prolonged storage. It has been shown that storage affects the stability of cytosolic protein complexes [17], and in an investigation on the storage-induced changes of the cytosolic red blood cell proteome, proteins were detected which could serve as potential markers for autologous blood doping [18, 19]. Other doping-related studies have looked into changes of the erythrocyte membrane proteome [20–22]. Currently, there is no information available on the specific impact of CPDA1 whole blood storage on the erythrocyte membrane proteome.

Therefore, this study is aimed at investigating changes in the erythrocyte membrane proteome of whole blood stored in CPDA1 blood bags. Liquid chromatography tandem mass spectrometry (LC-MS/MS), a method commonly used to investigate changes of the RBC proteome [23, 24], was used to identify possible changes that could potentially serve as markers to identify blood-doped athletes.

## 2. Materials and Methods

### 2.1. Materials

PlusOne Coomassie tablets PhastGel Blue R-350 and 12.5% ExcelGel were purchased from GE Healthcare Life Sciences (Uppsala, Sweden). CPDA1 blood bags were purchased from Medharmony (Qingdao, China). Dithiothreitol was purchased from Bio-Rad (Hercules, USA). Trypsin gold was purchased from Promega (Fitchburg, USA). Complete Protease Inhibitor Cocktail was purchased from Roche (Mannheim, Germany). Other chemicals and reagents were purchased from Sigma (Germany) unless indicated otherwise.

### 2.2. Subjects and Blood Collection

Six healthy female volunteers, aged 28–37 years, were recruited. All subjects were tested for their hemoglobin concentration, and only those with a concentration above 12 g/dL were included in the study. All participants provided written informed consent to the procedures approved by the ADLQ Institutional Review Board Ethics Committee (approval number: E20140000013). Age and hematological characteristics of the participants are shown in Table 1.

Subjects' heart rate, body temperature, and systolic and diastolic blood pressures were recorded before blood withdrawal and found to be in acceptable ranges. One unit of blood (450 mL ± 10%) was collected in CPDA1 blood bags, without buffy coat, plasma removal, or leukocyte filtration according to standard procedures at the Blood Donation Unit at Hamad Medical Corporation, Doha, Qatar. Blood cold chain was maintained for the transportation of blood bags from the blood donation center to the laboratory in order to avoid potential adverse effects by a temperature increase [25]. Blood bags were stored at 4 ± 2°C and 50 mL of whole blood was extracted from the bags under sterile conditions immediately after blood withdrawal and after 14 and 35 days of storage.

### 2.3. Preparation of Erythrocyte Ghosts

Peripheral blood mononuclear cells, platelets, and plasma were separated using Histopaque-1077, and the isolated erythrocytes were treated for 15 min at room temperature with 100 mM N-ethylmaleimide (NEM) in 20 mM phosphate buffer to quench free thiols prone to oxidation during protein isolation. This was followed by hypotonic lysis of erythrocytes with 5 mM phosphate buffer on ice for 20 min. Erythrocyte membranes (“ghosts”) were pelleted by centrifugation at 17000*g* for 20 min at 4°C. Erythrocyte ghosts were washed 3 times (17000*g*, 20 min, 4°C) with 5 mM phosphate buffer supplemented with 100 mM NEM and 1 tablet/50 mL Complete Protease Inhibitor Cocktail, followed by 3 washes with PBS (17000*g*, 20 min, 4°C). Samples were stored at −80°C until further analysis.

### 2.4. Preparation of Proteomic Samples

Erythrocyte ghost samples were centrifuged at 17000*g* for 20 min at 4°C. The pellet was resuspended in 4% SDS/0.1 M HEPES and incubated for 30 min at 37°C with agitation 1000 rpm. Protein concentration was determined with the Lowry assay using BSA as protein standard [26]. Normalized samples were applied to 12.5% ExcelGels and run on a Multiphor II Electrophoresis System (Amersham Biosciences) at 600 V, 30 mA, and 30 W for 140 min. Proteins were visualized by staining the gel with Coomassie PhastGel Blue R-350. The whole sample lines were divided into 15 equal parts, excised by hand, and placed in protein LoBind Eppendorf microcentrifuge tubes (Eppendorf, Hamburg, Germany). Excised gel pieces were destained overnight and washed for 2 hours with 50% MeOH in water containing 5% acetic acid. Gel pieces were then completely dehydrated with 100% acetonitrile in a vacuum centrifuge (Eppendorf, Hamburg, Germany) for 5 min. Subsequently, samples were reduced with 10 mM dithiothreitol in 100 mM ammonium bicarbonate and alkylated with 100 mM iodoacetamide in 100 mM ammonium bicarbonate. Finally, proteins were digested overnight at 37°C using 0.6 *μ*g of trypsin gold in 50 mM ammonium bicarbonate. Peptides were eluted in 50 mM ammonium bicarbonate with 0.1% formic acid, and the volume was reduced to 20 *μ*L using a vacuum centrifuge as described above.

### 2.5. Mass Spectrometry

Shotgun peptide mixtures were separated on a 25 cm reversed-phase C18 column (75 *μ*m, 2 *μ*m Acclaim RSLC C18, Thermo Scientific, Waltham, USA) using Easy n-LC II (Thermo Scientific, Waltham, USA) coupled to an Orbitrap Elite mass spectrometer (Thermo Scientific, Waltham, USA) over 68 min gradient of 5% to 60% acetonitrile (0.1% formic acid) with a constant flow of 300 nL/min. Thermo Xcalibur (version 3.0) software was used to control the instrument setup. Data-dependent acquisition (DDA) was performed using FTMS master scan preview mode, mass range 400–1800 *m*/*z* at 120000 resolution, for triggering MS/MS events. The mass spectra analyzed in DDA were set for charge state ≥ 2 and dynamic exclusion with 45-second duration for the most intense ion. Selected peptides were fragmented with collision-induced dissociation (CID) fragmentation with 2 *m*/*z* isolation window, normalized collision energy 35, and 10 ms activation time.

### 2.6. Data Processing and Analysis

Acquired MS/MS spectra were searched against UniProt *Homo sapiens* database (downloaded on 17 January 2017) using SEQUEST HT search engine in Proteome Discoverer 1.4 (PD 1.4, Thermo Scientific, San Jose, California, USA) to reveal the changes in the erythrocyte membrane proteome. The search parameters for CID-ion trap mass spectrometry (ITMS) fragment ion masses included 10 ppm precursor mass tolerances, 0.6 Da fragment mass tolerance, and maximum of 2 missed cleavages for tryptic peptides. A minimum precursor mass was set to 350 Da and the maximum to 5000 Da. The peptide false discovery rate (FDR) was calculated by a target-decoy approach and was set to 0.01. For protein identification, the following identification criteria were set: (1) at least 2 unique peptides and (2) XCorr value of at least 1.5 for singly, 2.0 for doubly, and 2.5 for triply charged peptides.

Furthermore, data acquired was also searched through the database using the comprehensive workflow that included several variable modifications of peptides (see Table S1), including carbamidomethylation at cysteine (+57.021 Da), N-terminal carboxymethyl and C-terminal oxidation and terminal-independent carbonylation (+13.979 Da), monooxidation (+15.995 Da), dioxidation (+31.990 Da), trioxidation (+47.985 Da), oxidation of His to Asn (−23.016 Da) or Asp (−22.032 Da), oxidation of Lys to aminoadipic acid (+14.963 Da), and carbonylation of Arg to glutamic semialdehyde (GluSA, −43.053 Da), Lys to aminoadipic semialdehyde (allysine, −1.032 Da), and Pro to pyrrolidinone (−30.010 Da) [27].

Only peptides with high confidence (*p* < 0.01) were used for filtering of the data for comparative proteomics and redox proteomics analysis of erythrocyte membrane proteins. Peptides with medium (*p* < 0.05) and high confidence (*p* < 0.01) were used for the initial filtering of data for label-free quantitation analysis.

The Software Tool for Rapid Annotation of Proteins (STRAP, version 1.5.0.0) developed by the Cardiovascular Proteomics Center, Center for Biomedical Mass Spectrometry at Boston University School of Medicine, was used to analyze gene ontology annotations available in the UniProt database [28].

### 2.7. Quantitative Statistical Analysis—Bayesian Approach

The analysis was performed on the values obtained with PD 1.4, described in the previous section, and the method further discussed was applied on peptides and proteins separately.

**Figure 1:**
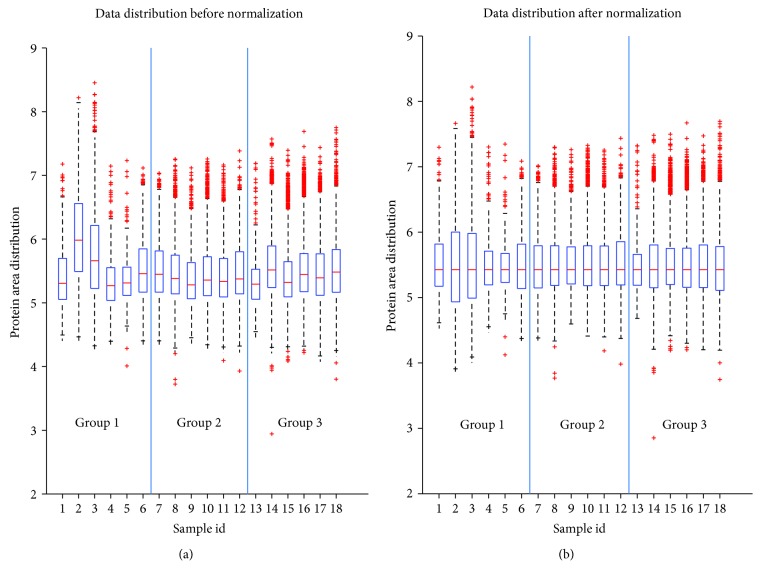
The distribution of the data before (a) and after (b) median scale normalization.

Prior statistical analysis data were preprocessed including the following criteria: (1) one set of variable (protein) for each subject, (2) only the variables that are present in over 5/6 subjects in at least one group, (3) exclusion of irrelevant variables, and (4) normalization using median scale normalization. To account for outliers (i.e., variability between the subjects in the group), we applied the median scale normalization described in [29]. Briefly, the data of each subject was multiplied by the ratio between its median and the entire data set median (of nonzero values). This is only valid for the assumption that the majority of the variables will not change and, implicitly, the groups should have the same median.  Figure 1(a) indicates the boxplot of the raw data, corresponding to each subject per group. Subjects 2 and 3 in group 1 (Figure 1(a) id, 2 and 3) have overall higher values of the areas; however, after the normalization (Figure 1(b)), the median for each subject is the same which indicates the reduction of the outliers.

As the study was conducted on the samples originating from the same 6 subjects differing in the storage duration, the statistical methods that account for the dependencies between groups have been applied. To assess the statistically significant differences between the “*fresh*” (i.e., group 1, day 0) and “*stored*” (i.e., groups 2 and 3, days 14 and 35, respectively) samples, the paired differences between the values of the variables (areas) were calculated based on the dependent set of observations according to the hypothesis:

(1) $$\begin{array}{c} H0_{i}:E\left(\Delta_{j-1,i}\right)=0,\\ H1_{i}:E\left(\Delta_{j-1,i}\right)\neq 0. \end{array}$$

*H*0 is the hypothesis that there are no significant changes before storage and after/during storage while *H*1 is the hypothesis that there are significant changes before storage and after/during storage. *E*(Δ_*j*−1,*i*_) represents the expected value (i.e., average) of the pairwise differences between the area in group *j* and group 1 (reference group day 0) for the same subject, while index *i* represents each variable for which the hypothesis testing was run.

In order to outline the variables (i.e., proteins and/or peptides) that provide sufficient evidence of storage-induced changes of the human erythrocyte membrane proteome, the quantitative proteome data was tested for normality using the Jarque-Bera test [30]. The test was performed per variable (protein/peptides) for each group separately and it showed that approximately 50% of the variables were not normally distributed. This indicates that a proper test would be a nonparametric test such as Wilcoxon. As the deviation from normality can be due to biological variability or outliers, a more robust test such as Bayesian hypothesis testing was also performed [31]. Pairwise comparison was applied on the differences Δ_*j*−1,*i*_ expressed in the previous equation. The core of the Bayesian statistics is the use of the following equation:

(2) $$\begin{array}{c} p\left(H_{i}\;|\;D\right)=\frac{p\left(D\;|\;H_{i}\right)p\left(H_{i}\right)}{\sum_{i}p\left(D\;|\;H_{i}\right)p\left(H_{i}\right)}, \end{array}$$

where *p*(*H*_*i*_ | *D*) is the posterior probability of the hypothesis *i* and *p*(*D* | *H*_*i*_) is the likelihood of the data given the hypothesis *H*_*i*_. In the previous equation, *p*(*H*_*i*_) is the prior probability of the hypothesis *H*_*i*_. The denominator of (2) is the normalization parameter. In the context of the hypothesis testing, two posterior probabilities of the competing hypotheses are compared as follows:

(3) $$\begin{array}{c} \frac{p\left(H_{1}\;|\;D\right)}{p\left(H_{0}\;|\;D\right)}=\frac{p\left(D\;|\;H_{1}\right)p\left(H_{1}\right)}{p\left(D\;|\;H_{0}\right)p\left(H_{0}\right)}. \end{array}$$

When no prior knowledge is available on each of the hypothesis, equal probability is assigned (i.e., 0.5); thus, the prior term in (3) is cancelled. The likelihood in this case is assumed to be Student's *t* distribution as it allows to include outliers without affecting the first moment (i.e., the mean). However, an integration is required over all the parameters of the distribution (mean, standard deviation, and degrees of freedom).

The posterior probability cannot be analytically integrated; hence, the sampling from the posterior must be employed (i.e., random samples are drawn for each parameter and are introduced in the expression of the posterior distribution, which is finally numerically integrated). For the sampling, the Metropolis-Hastings algorithm was used. For more details on this method, the reader is advised to address to the reference [24]. The Bayes factor, i.e., the posterior odds of the null hypothesis with respect to the alternative hypothesis, was estimated for tests of mean as a ratio between the prior to posterior probabilities in a region of practical equivalence (ROPE) according to Kruschke [31], with defined ROPE being equal to ±2.5% of the variable. A Bayes factor below 1 will indicate that the posterior odds are in favor of the alternative hypothesis (i.e., there is a significant difference), while a Bayes factor greater than 1 would mean the opposite.

Sample preparation steps can introduce spontaneous (artificial) methionine oxidation. The interference of artificial methionine oxidation has been minimized by simultaneous processing of all samples assuming that spontaneous oxidation of amino acids took place in all samples to the same or a highly similar extent. To quantify the ratios of oxidized versus nonoxidized (oxidized/nonoxidized) peptides [32], the peptide ion intensities were extracted from the MS/MS files of representative samples using precursor ion area detector node in PD 1.4. By combining the methionine oxidation results across all the samples, the doubtful oxidized methionine peptides were excluded from the data presentation. The degree of oxidation was measured using the ratio (oxidized/nonoxidized) peak areas of the same peptide in oxidized and nonoxidized form, respectively. Multivariate analysis (i.e., principal component analysis (PCA)) was applied to a selection of oxidized peptides in order to assess the clustering of the peptides for each day group [33].

An in-house script was built using MATLAB R2016B for the preprocessing (i.e., normality test and Wilcoxon and Bayesian hypothesis testing) of the data. All simulations were executed on a system with the following specifications: Intel(R) core(TM) i-7 6700 K CPU @ 4GHz, 64 G of RAM, 64 bit Windows 10 operating system.

## 3. Results

Changes in erythrocyte membrane proteome of human erythrocytes stored in CPDA1 bags were established by a comparative analysis of proteins identified in all fresh samples (*n* = 6) versus those identified in all stored samples, irrespective of the duration of storage (*n* = 12 with 6 for 14 and 6 for 35 days of storage) when analyzed for differentially expressed proteins and the redox proteome. Changes in the amount of membrane proteins were established by a comparative analysis of proteins identified in more than 80% (15 out of 18 samples) fresh samples (*n* = 6) with those identified in samples stored for 14 (*n* = 6) and 35 days (*n* = 6). Erythrocyte membrane proteins did not appear to be greatly different from fresh and stored samples on SDS gels (data not shown). This could be due to low resolution of SDS gels and because most abundant erythrocyte membrane proteins are either partially or fully retained in all samples irrespective of storage.

**Figure 2:**
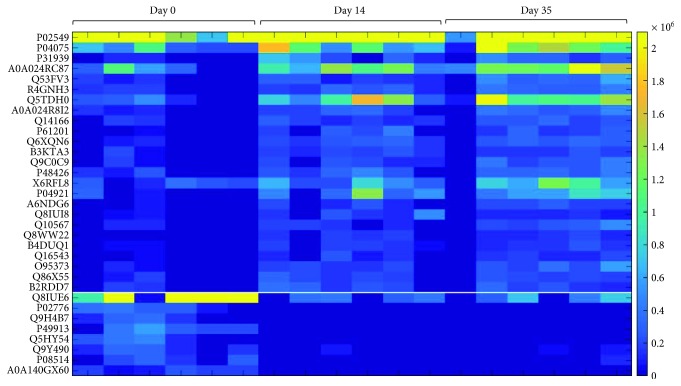
Heat map of differential proteins determined by the Bayesian test comparing day 0 to day 14 and day 0 to day 35 (FDR = 0.05). Protein ID description with fold change on the day 14 and day 35 is indicated in the Supplemental Table S2.

In contrast, the LC-MS/MS analysis of tryptic digests revealed significant storage time-dependent changes in the quantity of 33 proteins (Figure 2). The statistical evaluation of the data described in the “statistical evaluation” of the material and method section was applied to the proteins and peptides separately. The variables (i.e., peptides and proteins) were filtered in Bayesian framework as follows: if within the ROPE, the posterior odds are below 1, the variable is retained (i.e., there is sufficient evidence of a difference between the two likelihood distributions) otherwise the variable is excluded from the set of interest. The classical nonparametric approach, Wilcoxon test, filtered the variables that have a *p* value greater than 0.05, i.e., there is a significant evidence in favor of the null hypothesis (i.e., there is no difference between the concentrations from one day to another). The Wilcoxon test points to a significant change without discriminating between quantitative increase and decrease of the variable. The Bayesian approach however provides a posterior distribution from which an estimation of the fold change can be extracted by calculating the maximum a posteriori (MAP).

Statistical analysis includes common compounds that change in the same direction between starting date and day 14 and starting date and day 35. Supplemental Tables S2 and S3 include the quantitative fold change for the Bayesian statistical analysis of the peptides and proteins, respectively. The Wilcoxon test includes several variables (i.e., proteins and peptides) that are not highlighted by the Bayesian hypothesis test. This can be explained by the influence of the outliers on the hypothesis testing. The list of proteins identified by both approaches is presented in Supplemental Table S4. Although the increase or decrease in the quantity was in agreement with all peptides belonging to the same peptide, we have observed different fold changes with distinct peptides from the same protein (Supplemental Table S3). This is due to limitations of the applied mass spectrometry technique, as the same is not entirely quantitative due to different physiochemical properties of proteolytic peptides [34]. This can be overcome by comparing each individual peptide between different experimental groups. The quantitative analysis rendered 5 proteins involved in the erythrocyte metabolism, 3 proteins involved in erythrocyte membrane vesiculation, and 4 proteins involved in protein metabolism to be affected with storage. Furthermore, the presence of cytoplasmic proteins was observed in the stored erythrocyte membrane samples, such as adenylosuccinate lyase and flotillin-2.

**Figure 3:**
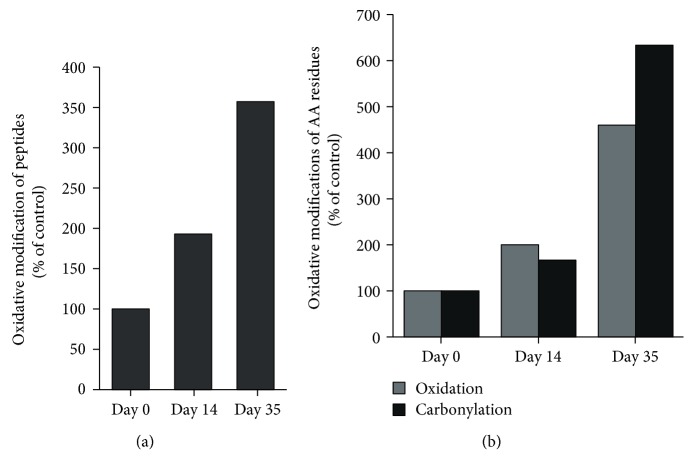
The impact of storage on (a) the number of oxidized peptides and (b) the type of oxidative modification of amino acid (AA) residues.

**Figure 4:**
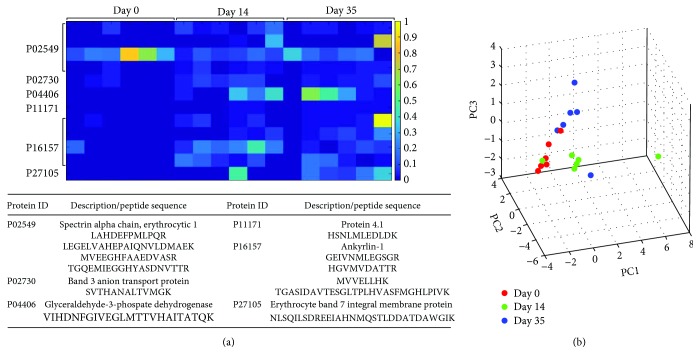
The heat map of semiquantitative assessment of peptide oxidations determined by Bayesian testing comparing day 0 to day 14 and day 0 to day 35 (FDR = 0.05): (a) protein ID description and peptide sequence are indicated in the table below the heat map; (b) PCA scatter plot showing the difference in the groups due to the oxidation.

The effect of storage on oxidation of erythrocyte membrane proteins is presented in Table 2 and Figures 3 and 4.

The redox proteomics analysis identified significant storage time-dependent oxidative stress-related posttranslational modifications (PTMs) of 14 membrane-associated proteins (PTMs) (Table 2). Storage promoted oxidation of the three glycolysis proteins, phosphoglycerate kinase 1 (PGK1), fructose-bisphosphate aldolase A, and glyceraldehyde-3-phosphate dehydrogenase (GAPDH). Moreover, Band 3 anion transport protein and erythrocyte structural proteins, spectrin alpha and beta chains, were also found to be prone to storage-induced oxidation.

A 3.5-fold increase in the number of oxidatively modified peptides was observed upon 35 days of storage (Figure 3(a)). Furthermore, the data show a storage-dependent increase in both oxidation and carbonylation of amino acid (AA) residues (Figure 3(b)). Though there was not a strong difference in the carbonylation of AA in samples stored for 14 days, the same showed a sixfold increase in the erythrocyte samples stored for 35 days (Figure 3(b)). As oxidations can be reversible modifications, it is possible that an increase in irreversible modifications may contribute to potential adverse posttransfusion outcomes.

Further analysis of quantitative changes in the ratios of oxidized versus nonoxidized (oxidized/nonoxidized) peptides are shown in Figure 4. A strong storage-dependent increase in (oxidized/nonoxidized) peptides was observed for peptides corresponding to GAPDH and Ankyrin-1 protein (Figure 4(a)). Finally, the multivariate analysis using PCA, on the oxidized peptides of samples between the three day groups, is shown on Figure 4(b). The figure shows the projections of the data on the first, second, and third principal components (scores plot). Close proximity of samples would indicate similar profiles; however, the results obtained showed a clear separation, storage-dependent clustering of samples between the three day groups.

**Figure 5:**
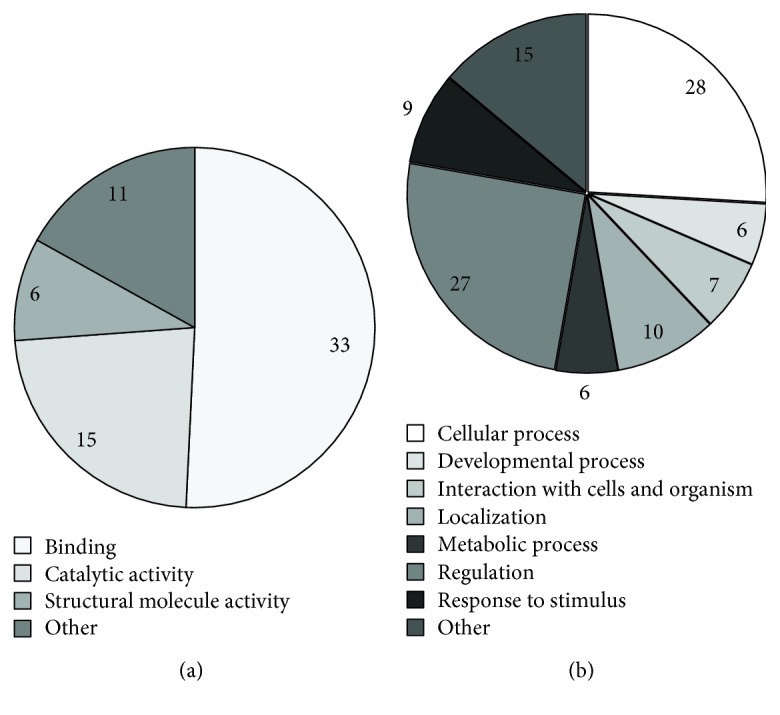
Repartition of molecular functions (a) and biological processes (b) corresponding to erythrocyte membrane proteins altered with the storage process.

All proteins found to be affected with the storage were investigated for their known functions reported in UniProt using STRAP software (Figure 5).

The obtained data show that the molecular function of the majority of the altered proteins identified by comparative proteomics and redox proteome analysis is related to binding activity, 73% (Figure 5(a)). Moreover, the majority of affected proteins identified by comparative proteomics analysis and redox proteome analysis are involved in cell regulation (60%, respectively), cellular processes (62%), and subcellular localization (22%) (Figure 5(b)).

## 4. Discussion

The LC-MS/MS analysis of the erythrocyte membrane proteome identified several proteins that were not identified in a single fresh sample from day 0 but were ubiquitously present in all stored samples. As erythrocytes cannot synthesize new proteins, this could be attributed to the migration of cytosolic proteins to the membrane triggered by storage-induced oxidative stress [35]. Indeed, some of the erythrocyte cytosolic proteins identified in all stored samples include adenylosuccinate lyase and flotillin-2. Migration of proteins in aging erythrocytes is a valuable tool to evaluate the quality of stored blood but also represents potentially good markers for the detection of autologous blood doping for which currently no direct detection method exists [36]. Quantitative analysis of proteins present in all samples identified 5 proteins involved in cell metabolism, including proteins involved in RBC energy production like fructose bisphosphate-aldolase A, glycerol-3-phosphate phosphatase, and nicotinate phosphoribosyltransferase, whose quantity increased in the stored samples. Storage not only impairs cellular energy production but it also affects erythrocyte membrane vesiculation [37]. This study indeed identified 3 proteins involved in the vesicle-mediated transport to be present in bigger amounts in the stored samples. On the other hand, 4 out of 11 proteins involved in protein metabolism, like tubulin beta-1 and tubulin alpha 4A chains, were found to be reduced in the membranes of stored samples. This reduction could potentially be attributed to the storage of blood at 4°C, as demonstrated earlier [38].

Analysis of storage-induced oxidative stress PTMs of the proteins present in all samples irrespective of storage revealed that prolonged blood storage rendered 14 erythrocyte membrane proteins prone to oxidative modifications in a time-dependent manner. Altered redox homeostasis in end-stage renal disease patients was shown to affect erythrocyte morphology and to induce erythrocyte membrane proteome remodeling [39]. One of the most common modification in this study was found to be oxidized methionine. Beside cysteine, methionine is another sulfur-containing AA implicated in the antioxidant protection of proteins in order to preserve their structure and function [40]. Although oxidation of methionine can be reversed, this PTM may act as a regulatory switch. Accumulation of oxidized methionine as a function of the storage time indicates deleterious effects of storage-induced oxidative stress. The oxidized methionine form can inhibit the phosphorylation of adjacent amino acid sites, such as serine, threonine, or tyrosine, in proteins altering protein function [41, 42]. Furthermore, prolonged storage led to a significant increase in the irreversible modifications of AA indicating a longer time period required to activate this type of PTMs compared to methionine oxidation. Moreover, it is likely that by the de novo sequencing, additional modifications would be identified as search databases can only partially address the PTMs [43].

Prolonged storage has been reported to affect the glycolytic pathway rendering cells susceptible to storage-induced oxidative damage of proteins. Indeed, results revealed three key proteins in glycolysis that are prone to storage-induced oxidation. The oxidation of PGK1, fructose-bisphosphate aldolase A, and GAPDH could be responsible for decreased glycolytic pathway in the stored samples and contribute to further oxidative injury of membrane proteins. This is supported by recent findings of Reisz and colleagues that reported the storage-dependent oxidation of GAPDH functional amino acid residues result in the enzyme activity loss [44]. Reversible oxidation of GAPDH results in metabolic shift from glycolysis towards the pentose phosphate pathway to counteract oxidative stress [45]. In addition, in intact erythrocytes, aldolase and GAPDH bind with high affinity to cytoplasmic domain of Band 3 anion transport protein [46, 47]. Although, to the best of our knowledge, there are no studies showing the binding affinity of PGK1 to Band 3 protein, as an immediate GAPDH downstream enzyme in the glycolytic pathway, it is possible that PGK1 is another protein involved in the band 3-dependent glycolytic regulation. PGK1 is the first ATP-generating enzyme in the glycolytic pathway [48], and the oxidation of the same can alter enzyme function and ATP production in erythrocytes. The impaired ATP production of stored erythrocytes is associated with poor *in vivo* survival [49] and was reported to potentially contribute to severe posttransfusion outcomes such as respiratory failure [50]. This study further showed that Band 3 protein is prone to storage-induced oxidative injury that could further impair its regulatory function of glycolysis and binding of glycolytic enzymes as earlier reported for phosphorylation of amino acid residues of the same protein [47].

An earlier time-course study of SAGM-stored erythrocytes showed continuous biochemical and structural alterations from day 14 onwards [51], that is, in agreement with the CPDA1-stored erythrocytes (this study). Protein oxidation is one of the PTMs that affects organization of RBC complexes [52]. The oxidative modification of erythrocyte structural proteins, such as spectrin alpha and beta chains, identified in this study, has been reported in the literature to promote erythrocyte microparticle formation [53, 54]. Also, prolonged storage in CPDA was found to increase carbonylation of cytoskeletal proteins and accumulation of oxidized hemoglobin that could promote storage-induced vesiculation [55]. In a recent publication, using the same samples, we reported a 100-fold increase in erythrocyte microparticles already 14 days after storage [56]. Oxidative damage of band 3 and other membrane proteins triggers a series of events leading to vesiculation [57]. It has been proposed that in high oxidative stress, microenvironment oxidatively damaged proteins that accumulate in vesicles could represent a mechanism of removing damaged proteins [23, 58]. Indeed, an initial gradual increase throughout several weeks in the carbonylated RBC proteins decreases by the end of the storage that could be attributed to vesiculation [59].

Additionally, the multivariate PCA of oxidized peptides revealed clear clustering of the samples for each day group, indicating that the oxidized form of the same peptide has a higher intensity (and consequently higher area) of the peaks. Moreover, the fact that all 3 day groups are clustered (i.e., the cluster centroids) distant from one another points to the fact that the oxidation is different from day 0 to day 14 and to day 35.

Taken together, comparative proteomics, redox proteomics, and quantitative proteomics analyses revealed proteins that could become useful markers to identify athletes that have received an ABT. Still, the stability and lifetime of those markers posttransfusion remain unknown. The posttransfusion clearance of erythrocytes depends on the duration of storage and on the preservative used. Within the first hour posttransfusion, the majority of transfused damaged erythrocytes are cleared; however, still more than 75% of erythrocytes remain in the circulation 24 hours posttransfusion [60]. Interestingly, although CPDA1 is one of the standard preservatives, the leukoreduced CPDA1 erythrocytes were shown to contribute to harmful effects associated with blood transfusion [61] and it is likely that different changes might have occurred in the membranes of those erythrocytes. Thus, it is possible that different markers will rise from erythrocytes stored under different conditions including compositions of cellular components stored. Furthermore, the alterations of the erythrocyte membrane proteome might be responsible for the impaired erythrocyte energy and protein metabolism and vesiculation, contributing to potential adverse posttransfusion outcomes. Thus, the understanding of the posttransfusion consequences on the human health and erythrocyte fate needs to be elucidated. Altogether, the data demonstrate the importance of studying storage-induced alterations in erythrocyte membrane proteome and the need to understand the clearance kinetics of transfused erythrocytes and protein markers identified in this study.

## Figures and Tables

**Table 1:** Age and hematological characteristics of participants.

Parameter	Unit	Mean ± SD
Age	Years	32.17 ± 3.19
WBC	10^3^/*μ*L	7.77 ± 2.16
LYMPH	%	37.02 ± 8.37
MONO	%	8.13 ± 2.03
GRAN	%	54.85 ± 10.27
RBC	10^6^/*μ*L	4.95 ± 0.28
Hb	g/dL	13.30 ± 0.60
Hct	%	40.93 ± 1.39
PLT	10^3^/*μ*L	258.00 ± 49.09

WBC: white blood cell count; LYMPH: lymphocyte count; MONO: monocyte count; GRAN: granulocyte count; RBC: red blood cell count; Hb: hemoglobin concentration; Hct: hematocrit; PLT: platelet count.

**Table 2:** Oxidative modification of proteins ubiquitously present in samples irrespective of storage. Modifications labeled with Ox or C represent either peptide oxidation or peptide carbonylation, respectively.

Accession	Description	Sequence	Modification		
			Day 0	Day 14	Day 35
A0A0K2BMD8	Mutant hemoglobin alpha 2 globin chain	VADALTNAVAHVDD**M**PNALSALSDLHAHK		Ox	Ox
P00558	Phosphoglycerate kinase 1	AHSS**M**VGVNLPQK			Ox
		IQLINN**M**LDK		Ox	Ox
		SVVLMSHLGRPDGVP**M**PDK			Ox
		VNE**M**IIGGGMAFTFLK			Ox
P02042	Hemoglobin subunit delta	EFTPQ**M**QAAYQK	Ox		
P02549	Spectrin alpha chain, erythrocytic 1	AD**M**EA**E**A**P**TFQALEDFSAELIDSGHHASPEIEK	Ox, C	Ox	Ox
		EK**M**EILDNNWTALLELWDER		Ox	Ox
		FSSDFDELSGW**M**N**E**K	Ox	Ox, C	Ox
		LTLSHPSDA**P**Q**I**QE**M**KEDLVSSWEHIR			Ox, C
		SDDKSSLDSLEAL**M**K		Ox	Ox
P02730	Band 3 anion transport protein	GLDLNGGPDD**PL**QQTGQLFGGLVR			C
		YTQEIFSF**L**ISLIFIYETFSKLIKIFQDHPLQK			C
		PQGPLPNTALLSLVLMAGTFFFAM**M**LR		Ox	Ox
		RYQSSPAKPDSS**FY**K			Ox
P04075	Fructose-bisphosphate aldolase A	IGEHTPSALAI**M**ENANVLAR			Ox
P04406	Glyceraldehyde-3-phosphate dehydrogenase	WGDAGAEYVVESTGVFTT**M**EK			Ox
P11166	Solute carrier family 2, facilitated glucose transporter member 1	QGGASQSDKT**PE**ELFHPLGADSQV			C
		SFE**M**LILGR	Ox		
P11171	Protein 4.1	S**M**TPAQADLEFLENAK		Ox	Ox
P11277	Spectrin beta chain, erythrocytic	DEEGAIV**M**LK	Ox	Ox	
		DGLNE**M**WADLLELIDTR		Ox	Ox
		DLEDET**L**WVEER		C	C
		ESQQL**M**DSHPEQK			Ox
		GLDAHLEQIFQEAHG**M**VAR		Ox	Ox
		HQAFVA**E**LASH**E**GWLENIDAEGK		C	
		KEELGELFAQVPS**M**G**E**EGGDADLSIEK			Ox, C
		L**W**S**Y**LQELLQSR		Ox	Ox
		QL**M**D**E**K**P**QFTALVSQK	C	C	Ox, C
		VISDEIPKDEEGAIV**M**LK			Ox
P16157	Ankyrin-1	DIEVLEG**M**SLFAELSGNLVPVKK			Ox
		EGQNAN**M**ENLYTALQSIDR			Ox
		GFTPLY**M**AAQENHLEVVK			Ox
		HGV**M**VDATTR		Ox	Ox
		**M**GYT**PL**HVASHYGNIK**L**VK			Ox, C
		SLLQYGGSANAESVQGVT**P**LHLAAQEGHAE**M**VAL**L**LSK			Ox, C
		TGASIDAVTESGLTPLHVASF**M**GHLPIVK		Ox	Ox
		VETPLH**M**AAR		Ox	
P27105	Erythrocyte band 7 integral membrane protein	EEIAH**N**MQSTLDDATDAWGIK			Ox
		NLSQILSDREEIAHN**M**QSTLDDATDAWGIK		Ox	Ox
P52209-2	Isoform 2 of 6-phosphogluconate dehydrogenase, decarboxylating	YGPSL**M**PGGNK			Ox
P60709	Actin, cytoplasmic 1	KDLYANTVLSGGTT**M**YPGIADR			Ox

## Data Availability

The proteomics data used to support the findings of this study are available from the corresponding author upon request.

## Supplementary Materials

Supplementary 1 Table S1: list of all oxidative modifications searched.

Supplementary 2 Table S2: quantitative fold change for Bayesian statistical analysis of proteins.

Supplementary 3 Table S3: quantitative fold change for Bayesian statistical analysis of peptides. The minimum and maximal XCorr values for each peptide determined in samples are included in the table.

Supplementary 4 Table S4: quantitative fold change of proteins commonly identified for Bayesian and Wilcoxon statistical analyses.
