# Supplementary material for: Whole Blood Storage in CPDA1 Blood Bags Alters Erythrocyte Membrane Proteome
Source: Oxid Med Cell Longev. 2018 Nov 8;2018:6375379. doi: 10.1155/2018/6375379 (PMC6249999; doi:10.1155/2018/6375379)
Supplement: Supplementary 1 — Table S1: list of all oxidative modifications searched. [file 6375379.f1.docx]

**Table S1.** List of all oxidative modifications searched.

| **Modification** | **Mass Change  (Da)** | **AA Residues** |
| --- | --- | --- |
| Monooxidation | 15.995 | C, D, F, H, K, M, N, W, Y |
| Dioxidation | 31.990 | C, F, K, M, P, R, W, Y |
| Trioxidation | 47.985 | C, W, Y |
| C-terminal oxidation | 15.995 | Any |
| Carbonylation | 13.979 | E, I, K, L, P, Q, R |
| Lys - > Allysine | 1.032 | K |
| Arg -> GluSA | 43.053 | R |
| Pro -> Pyrrolidinone | 30.011 | P |
| His -> Asn | 23.016 | H |
| His -> Asp | 22.032 | H |
| Lys -> AminoadipicAcid | 14.963 | K |
