# Supplementary material for: Whole Blood Storage in CPDA1 Blood Bags Alters Erythrocyte Membrane Proteome
Source: Oxid Med Cell Longev. 2018 Nov 8;2018:6375379. doi: 10.1155/2018/6375379 (PMC6249999; doi:10.1155/2018/6375379)
Supplement: Supplementary 2 — Table S2: quantitative fold change for Bayesian statistical analysis of proteins. [file 6375379.f2.docx]

**Table S2.** Quantitative fold-change for Bayesian statistical analysis of proteins.

| **Bayesian hypothesis testing** | |  |  |
| --- | --- | --- | --- |
| **Increasing quantitatively** | | **Fold change after 14 days** | **Fold change after 35days** |
| **Accession ID** | **Description** |  |  |
| P02549 | Spectrin alpha chain, erythrocytic 1 | 3.46 | 2.61 |
| P04075 | Fructose-bisphosphate aldolase A | 2.08 | 2.78 |
| P31939 | Bifunctional purine biosynthesis protein PURH | 6.92 | 6.47 |
| A0A024RC87 | Ribonuclease/angiogenin inhibitor 1 | 2.74 | 3.28 |
| Q53FV3 | COP9 signalosome subunit 4 variant (Fragment) | 2.73 | 5.31 |
| R4GNH3 | 26S protease regulatory subunit 6A | 2.71 | 3.70 |
| Q5TDH0 | Protein DDI1 homolog 2 | 4.52 | 6.01 |
| A0A024R8I2 | Ubiquitin associated domain containing 1 | 3.19 | 5.34 |
| Q14166 | Tubulin--tyrosine ligase-like protein 12 | 3.17 | 3.58 |
| P61201 | COP9 signalosome complex subunit 2 | 28.58 | 29.14 |
| Q6XQN6 | Nicotinate phosphoribosyltransferase | 6.80 | 7.88 |
| B3KTA3 | Fascin | 5.03 | 3.68 |
| Q9C0C9 | (E3-independent) E2 ubiquitin-conjugating enzyme | 5.26 | 6.97 |
| P48426 | Phosphatidylinositol 5-phosphate 4-kinase type-2 alpha | 2.32 | 3.57 |
| X6RFL8 | Ras-related protein Rab-14 (Fragment) | 2.00 | 3.39 |
| P04921 | Glycophorin-C | 5.65 | 9.07 |
| A6NDG6 | Glycerol-3-phosphate phosphatase | 7.80 | 14.15 |
| Q8IUI8 | Cytokine receptor-like factor 3 | 7.91 | 4.28 |
| Q10567 | AP-1 complex subunit beta-1 | 2.86 | 6.43 |
| Q8WW22 | DnaJ homolog subfamily A member 4 | 25.09 | 25.61 |
| B4DUQ1 | cDNA FLJ54552, highly similar to Heterogeneous nuclear ribonucleoprotein K | 7.16 | 7.41 |
| Q16543 | Hsp90 co-chaperone Cdc37 | 18.12 | 24.88 |
| O95373 | Importin-7 | 6.37 | 9.01 |
| Q86X55 | Histone-arginine methyltransferase CARM1 | 3.74 | 3.77 |
| B2RDD7 | Protein arginine N-methyltransferase 5 | 4.56 | 3.57 |
|  |  |  |  |
| **Bayesian hypothesis testing** | |  |  |
| **Decreasing quantitatively** | | **Fold change after 14 days** | **Fold change after 35days** |
| **Accession ID** | **Description** |  |  |
| Q8IUE6 | Histone H2A type 2-B | 0.20 | 0.30 |
| P02776 | Platelet factor 4 | 0.04 | 0.04 |
| Q9H4B7 | Tubulin beta-1 chain | 0.01 | 0.05 |
| P49913 | Cathelicidin antimicrobial peptide | 0.08 | 0.01 |
| Q5HY54 | Filamin-A | 0.05 | 0.05 |
| Q9Y490 | Talin-1 | 0.15 | 0.23 |
| P08514 | Integrin alpha-Iib | 0.06 | 0.04 |
| A0A140GX60 | Platelet membrane glycoprotein Ib beta | 0.06 | 0.06 |
