# Supplementary material for: Whole Blood Storage in CPDA1 Blood Bags Alters Erythrocyte Membrane Proteome
Source: Oxid Med Cell Longev. 2018 Nov 8;2018:6375379. doi: 10.1155/2018/6375379 (PMC6249999; doi:10.1155/2018/6375379)
Supplement: Supplementary 3 — Table S3: quantitative fold change for Bayesian statistical analysis of peptides. The minimum and maximal XCorr values for each peptide determined in samples are included in the table. [file 6375379.f3.docx]

**Table S3**. Quantitative fold-change for Bayesian statistical analysis of peptides. The minimum and maximal XCorr values for each peptide determined in samples is included in the table.

| **Protein ID** | **Protein name and Peptide Sequence** | **Fold-Change day 14** | **Fold-Change day 35** | **Min XCorr** | **Max XCorr** |
| --- | --- | --- | --- | --- | --- |
| **A0A024R8I2** | **Ubiquitin associated domain containing 1, isoform CRA_c** | |  |  |  |
|  | AVISLMEMGFDEK | 7.44 | 15.37 | 2.93 | 4.68 |
|  | LLALNPDAVELFK | 4.39 | 7.39 | 2.81 | 4.70 |
| **A0A024RC87** | **Ribonuclease/angiogenin inhibitor 1, isoform CRA_a** | |  |  |  |
|  | LDDCGLTEAR | 12.11 | 18.70 | 2.45 | 3.89 |
|  | VNPALAELNLR | 5.81 | 9.31 | 3.16 | 4.38 |
| **A0A087WUQ6** | **Glutathione peroxidase** |  |  |  |  |
|  | FLVGPDGVPLR | 19.26 | 39.89 | 2.61 | 3.85 |
| **A0A0K2BMD8** | **Mutant hemoglobin alpha 2 globin chain** |  |  |  |  |
|  | KVADALTNAVAHVDDMPNALSALSDLHAHK | 7.26 | 15.82 | 3.13 | 6.58 |
|  | VADALTNAVAHVDDMPNALSALSDLHAHK | 4.47 | 5.01 | 3.48 | 6.31 |
| **A0A140GX60** | **Platelet membrane glycoprotein Ib beta** |  |  |  |  |
|  | LLPYLAEDELR | 0.07 | 0.08 | 2.91 | 3.22 |
| **A0A1B0GTJ7** | **Adenylosuccinate lyase** |  |  |  |  |
|  | SNLENIDFK | 10.01 | 15.60 | 2.38 | 3.19 |
|  | VLSQQAASVVK | 5.14 | 6.44 | 2.28 | 4.29 |
| **A0A1B0GWG0** | **Protein 4.1 (Fragment)** |  |  |  |  |
|  | SLDGAAVDSADR | 5.66 | 9.24 | 3.28 | 4.28 |
| **A6NDG6** | **Glycerol-3-phosphate phosphatase** |  |  |  |  |
|  | TILTLTGVSTLGDVK | 21.07 | 61.39 | 3.31 | 5.51 |
| **A8K486** | **Peptidyl-prolyl cis-trans isomerase** |  |  |  |  |
|  | VSFELFADK | 6.49 | 14.25 | 2.68 | 3.57 |
| **A8K4T6** | **cDNA FLJ76282, highly similar to Homo sapiens proteasome (prosome, macropain) 26S subunit, non-ATPase, 5 (PSMD5), mRNA** | | | | |
|  | LLQAMEPVHVAR | 14.77 | 25.05 | 2.67 | 3.55 |
|  | TIAEIFGNPNYLR | 14.86 | 27.99 | 3.43 | 4.64 |
| **A8K607** | **cDNA FLJ76855, highly similar to Homo sapiens exportin 7 (XPO7), mRNA** | | | |  |
|  | ILTLGEVPK | 7.42 | 10.77 | 2.50 | 3.05 |
|  | SNYQLGELVK | 27.30 | 42.53 | 2.66 | 3.24 |
| **A8K6Y1** | **cDNA FLJ75526, highly similar to Homo sapiens proliferation-associated 2G4, 38kDa (PA2G4), mRNA (Fragment)** | | | | |
|  | SDQDYILK | 3.77 | 8.60 | 2.91 | 3.56 |
| **A8K8U1** | **cDNA FLJ77762, highly similar to Homo sapiens cullin-associated and neddylation-dissociated 1 (CAND1), mRNA** | | | | |
|  | AADIDQEVK | 3.65 | 5.73 | 2.63 | 3.21 |
|  | AVAALLTIPEAEK | 14.04 | 29.81 | 2.85 | 4.33 |
|  | MLTGPVYSQSTALTHK | 12.11 | 28.77 | 2.86 | 4.08 |
| **B2R983** | **cDNA, FLJ94267, highly similar to Homo sapiens glutathione S-transferase omega 1 (GSTO1), mRNA** | | | | |
|  | EDPTVSALLTSEK | 20.81 | 50.18 | 2.45 | 3.93 |
| **B2RBR9** | **cDNA, FLJ95650, highly similar to Homo sapiens karyopherin (importin) beta 1 (KPNB1), mRNA** | | | | |
|  | LAATNALLNSLEFTK | 6.12 | 13.90 | 3.96 | 5.99 |
|  | WLAIDANAR | 11.34 | 25.32 | 2.51 | 3.12 |
| **B2RD79** | **cDNA, FLJ96494, highly similar to Homo sapiens ubiquitin specific peptidase 14 (tRNA-guanine transglycosylase) (USP14), mRNA** | | | | |
|  | RVEIMEEESEQ | 2.91 | 6.44 | 2.95 | 4.04 |
| **B2RDD7** | **Protein arginine N-methyltransferase 5** |  |  |  |  |
|  | YSQYQQAIYK | 13.78 | 12.50 | 3.00 | 4.41 |
| **B3KNN7** | **cDNA FLJ30049 fis, clone ADRGL1000033, highly similar to 26S proteasome non-ATPase regulatory subunit 3** | | | | |
|  | AIQLEYSEAR | 3.80 | 6.15 | 2.56 | 4.29 |
| **B3KTA3** | **Fascin** |  |  |  |  |
|  | YLTAEAFGFK | 5.56 | 8.43 | 2.61 | 3.73 |
| **B3KX11** | **T-complex protein 1 subunit gamma** |  |  |  |  |
|  | IVLLDSSLEYK | 10.44 | 25.11 | 2.72 | 3.88 |
| **B4DDF7** | **cDNA FLJ53296, highly similar to Serine/threonine-protein phosphatase 2A 65 kDa regulatory subunit A alpha isoform** | | | | |
|  | IGPILDNSTLQSEVKPILEK | 20.08 | 27.48 | 3.02 | 4.85 |
|  | VLELDNVK | 8.17 | 24.47 | 2.39 | 2.81 |
| **B5BUB1** | **RuvB-like helicase (Fragment)** |  |  |  |  |
|  | GLGLDESGLAK | 7.15 | 14.11 | 2.62 | 3.68 |
| **E7EV99** | **Alpha-adducin** |  |  |  |  |
|  | VDENNPEYLR | 4.16 | 8.73 | 3.04 | 4.09 |
| **O00231** | **26S proteasome non-ATPase regulatory subunit 11** | |  |  |  |
|  | LQATLDMQSGIIHAAEEK | 15.30 | 31.50 | 3.41 | 4.92 |
| **O00232** | **26S proteasome non-ATPase regulatory subunit 12** | |  |  |  |
|  | LFTTMELMR | 7.60 | 14.87 | 2.71 | 3.71 |
| **O00299** | **Chloride intracellular channel protein 1** |  |  |  |  |
|  | YLSNAYAR | 11.76 | 28.71 | 2.29 | 2.75 |
| **O14980** | **Exportin-1** |  |  |  |  |
|  | LISGWVSR | 8.08 | 11.37 | 2.47 | 3.13 |
| **O75955** | **Flotillin-1** |  |  |  |  |
|  | GEAEAFAIGAR | 2.96 | 3.52 | 3.18 | 4.34 |
|  | ISLNTLTLNVK | 4.93 | 5.90 | 2.87 | 4.05 |
|  | KAEAFQLYQEAAQLDMLLEK | 10.45 | 10.99 | 4.35 | 6.00 |
|  | VQVQVVER | 3.91 | 4.17 | 2.31 | 3.10 |
|  | VSAQYLSEIEMAK | 3.46 | 7.16 | 3.59 | 5.00 |
| **O95373** | **Importin-7** |  |  |  |  |
|  | AFAVGVQQVLLK | 7.83 | 15.21 | 3.69 | 4.72 |
| **O95747** | **Serine/threonine-protein kinase OSR1** |  |  |  |  |
|  | SGVLDESTIATILR | 7.35 | 13.60 | 3.52 | 4.56 |
| **P00338** | **L-lactate dehydrogenase A chain** |  |  |  |  |
|  | VTLTSEEEAR | 5.44 | 7.81 | 2.99 | 3.63 |
| **P00558** | **Phosphoglycerate kinase 1** |  |  |  |  |
|  | ITLPVDFVTADK | 16.71 | 41.01 | 2.30 | 3.59 |
|  | LGDVYVNDAFGTAHR | 3.97 | 5.78 | 2.72 | 4.95 |
|  | YSLEPVAVELK | 4.00 | 11.79 | 2.49 | 3.81 |
| **P00568** | **Adenylate kinase isoenzyme 1** |  |  |  |  |
|  | ATEPVIAFYEK | 6.43 | 7.33 | 2.39 | 3.07 |
| **P02042** | **Hemoglobin subunit delta** |  |  |  |  |
|  | VNVDAVGGEALGR | 7.27 | 10.83 | 3.35 | 5.14 |
| **P02549** | **Spectrin alpha chain, erythrocytic 1** |  |  |  |  |
|  | AAPVEGVAGEQR | 11.21 | 11.67 | 3.18 | 4.27 |
|  | ADVVEAWIADK | 4.40 | 4.83 | 3.34 | 4.58 |
|  | ADVVEAWIADKETSLK | 3.05 | 3.92 | 2.67 | 5.72 |
|  | AENTGVELDDVWELQK | 10.99 | 9.55 | 3.04 | 5.60 |
|  | ALSNAANLQR | 4.88 | 4.31 | 3.24 | 3.99 |
|  | ASALNNWCEK | 6.62 | 8.55 | 2.50 | 3.56 |
|  | AYFLDGSLLK | 4.37 | 4.06 | 2.70 | 3.45 |
|  | DLNTLAEDLLSSGTFNVDQIVK | 4.85 | 4.00 | 4.65 | 7.64 |
|  | DLQGVQNLLK | 3.97 | 4.40 | 2.87 | 4.03 |
|  | DSEQVDSWMSR | 11.31 | 12.90 | 2.25 | 4.26 |
|  | DTEDEEAWIQETEPSATSTYLGK | 6.30 | 6.62 | 2.81 | 5.53 |
|  | DVTEAIQWIK | 5.97 | 7.74 | 3.31 | 4.36 |
|  | EAIATSVELGEDWER | 4.34 | 7.45 | 2.53 | 5.43 |
|  | EDLVSSWEHIR | 4.20 | 7.31 | 2.90 | 4.11 |
|  | EHWDHLLER | 8.43 | 7.25 | 2.51 | 3.27 |
|  | EKEPVLTSEDYGK | 4.21 | 4.64 | 2.68 | 4.39 |
|  | EKEPVLTSEDYGKDLVASEGLFHSHK | 22.98 | 15.42 | 3.02 | 5.38 |
|  | ETGTLESQLEANK | 4.24 | 3.35 | 3.03 | 4.36 |
|  | FLDAVDPGR | 5.46 | 4.56 | 2.43 | 3.63 |
|  | FYLFLSK | 4.73 | 4.46 | 2.25 | 2.90 |
|  | GDCGDTLAATQSLLMK | 5.59 | 9.33 | 2.37 | 5.42 |
|  | HEAFLLDLNSFGDSMK | 5.24 | 10.52 | 3.27 | 5.19 |
|  | HEIDSYDDR | 7.86 | 7.98 | 2.55 | 3.69 |
|  | HFDENLTGR | 5.61 | 6.09 | 2.62 | 3.71 |
|  | HGLLESAVAAR | 3.25 | 4.78 | 2.91 | 3.75 |
|  | ITDLEHFAESLIADEHYAK | 5.54 | 4.53 | 2.86 | 7.00 |
|  | IVDLGDNLEDALILDIK | 7.60 | 5.78 | 5.37 | 6.86 |
|  | KAENTGVELDDVWELQK | 4.50 | 7.64 | 3.35 | 5.79 |
|  | KFEDFQVELVAK | 3.99 | 4.48 | 4.26 | 5.72 |
|  | KGDVLTLLSSINK | 3.84 | 3.93 | 3.93 | 5.46 |
|  | KGYVSLEDYTAFLIDK | 2.60 | 3.20 | 3.73 | 6.56 |
|  | KHGLLESAVAAR | 2.95 | 6.54 | 2.88 | 4.17 |
|  | KLYEDSDDLK | 4.96 | 6.11 | 2.88 | 4.29 |
|  | LADDEDYKDIQNLK | 3.43 | 6.74 | 2.87 | 5.67 |
|  | LADLHEAETWIR | 2.95 | 7.07 | 3.37 | 4.56 |
|  | LAHDEFPMLPQR | 5.26 | 6.03 | 2.68 | 3.71 |
|  | LAQFVEHWEK | 5.67 | 5.22 | 3.29 | 4.07 |
|  | LEESLEYLQFMQNAEEEEAWINEK | 4.84 | 11.78 | 3.93 | 8.24 |
|  | LERDDLEK | 11.15 | 7.90 | 2.33 | 2.94 |
|  | LGDYANLK | 4.38 | 7.37 | 2.69 | 3.49 |
|  | LMSELEK | 6.56 | 9.10 | 2.37 | 2.67 |
|  | LSEVASLWEELLEATK | 6.82 | 7.51 | 3.25 | 6.10 |
|  | LTLSHPSDAPQIQEMK_O | 8.32 | 5.36 | 3.58 | 5.07 |
|  | LTLSHPSDAPQIQEMK | 4.67 | 5.59 | 4.06 | 5.03 |
|  | LYEDSDDLK | 6.35 | 6.31 | 2.32 | 3.25 |
|  | MEILDNNWTALLELWDER | 10.71 | 11.41 | 3.49 | 6.22 |
|  | MELNEAWEDLQGR_O | 9.43 | 5.88 | 3.53 | 4.84 |
|  | MELNEAWEDLQGR | 4.03 | 5.28 | 3.95 | 5.51 |
|  | MQHNLEQQIQAK | 4.85 | 4.14 | 3.50 | 5.18 |
|  | MVEEGHFAAEDVASR | 4.40 | 6.35 | 3.14 | 5.17 |
|  | NLAVMSDK | 3.76 | 7.89 | 2.45 | 2.89 |
|  | QEAFLENEDLGNSLGSAEALLQK | 11.88 | 27.47 | 3.04 | 7.20 |
|  | QLLGSAHAVEVFHR | 3.09 | 5.20 | 2.75 | 3.89 |
|  | SLGSAEALLQK | 17.99 | 19.37 | 3.07 | 3.95 |
|  | SSDEIENAFQALAEGK | 10.07 | 9.25 | 4.73 | 6.10 |
|  | SSLDSLEALMK | 4.90 | 4.51 | 3.20 | 4.13 |
|  | SYEDPTNIQGK | 3.37 | 2.92 | 3.20 | 4.13 |
|  | TGQEMIEGGHYASDNVTTR_O | 14.05 | 18.40 | 3.02 | 5.12 |
|  | TGQEMIEGGHYASDNVTTR | 5.31 | 9.27 | 3.42 | 5.61 |
|  | VADDLLFEGLLTPEGAQIR | 4.43 | 4.31 | 3.29 | 5.62 |
|  | VLETAEEIQER | 6.78 | 10.63 | 3.40 | 4.53 |
|  | VMALYDFQAR | 4.46 | 3.79 | 3.21 | 4.00 |
| **P02730** | **Band 3 anion transport protein** |  |  |  |  |
|  | ADFLEQPVLGF | 6.73 | 20.46 | 2.81 | 3.49 |
|  | APSEQALLSLVPVQR | 21.32 | 26.49 | 3.10 | 4.39 |
|  | GLDLNGGPDDPLQQTGQLFGGLVR | 2.59 | 3.85 | 3.72 | 6.56 |
|  | SVTHANALTVMGK_O | 7.81 | 8.45 | 2.95 | 5.03 |
|  | SVTHANALTVMGK | 2.60 | 4.94 | 3.72 | 5.34 |
| **P02776** | **Platelet factor 4** |  |  |  |  |
|  | AGPHCPTAQLIATLK | 0.09 | 0.18 | 3.14 | 4.09 |
| **P04075** | **Fructose-bisphosphate aldolase A** |  |  |  |  |
|  | AAQEEYVK | 4.88 | 9.16 | 2.75 | 3.36 |
|  | ALQASALK | 3.80 | 5.86 | 2.29 | 3.06 |
|  | GILAADESTGSIAK | 3.29 | 4.36 | 3.92 | 5.26 |
|  | IGEHTPSALAIMENANVLAR | 6.21 | 9.92 | 2.76 | 5.45 |
| **P04406** | **Glyceraldehyde-3-phosphate dehydrogenase** |  |  |  |  |
|  | VPTANVSVVDLTCR | 6.87 | 22.80 | 3.46 | 4.92 |
| **P05109** | **Protein S100-A8** |  |  |  |  |
|  | ALNSIIDVYHK | 0.03 | 0.13 | 2.64 | 3.74 |
| **P07195** | **L-lactate dehydrogenase B chain** |  |  |  |  |
|  | GLTSVINQK | 9.75 | 13.26 | 2.31 | 3.09 |
|  | SADTLWDIQK | 11.87 | 27.02 | 2.81 | 3.86 |
| **P07384** | **Calpain-1 catalytic subunit** |  |  |  |  |
|  | DFFLANASR | 7.15 | 9.08 | 2.67 | 3.57 |
|  | LVFVHSAEGNEFWSALLEK | 10.94 | 13.67 | 3.34 | 4.46 |
|  | SEQFINLR | 13.83 | 21.70 | 2.44 | 2.94 |
| **P07900** | **Heat shock protein HSP 90-alpha** |  |  |  |  |
|  | DQVANSAFVER | 3.41 | 6.03 | 2.67 | 4.04 |
|  | ELISNSSDALDK | 2.98 | 6.78 | 2.88 | 4.00 |
|  | YYTSASGDEMVSLK | 5.43 | 6.74 | 3.06 | 5.51 |
| **P08311** | **Cathepsin G** |  |  |  |  |
|  | VSSFLPWIR | 0.15 | 0.26 | 2.55 | 2.80 |
| **P08514** | **Integrin alpha-IIb** |  |  |  |  |
|  | VAIVVGAPR | 0.14 | 0.21 | 2.63 | 2.93 |
| **P11142** | **Heat shock cognate 71 kDa protein** |  |  |  |  |
|  | DAGTIAGLNVLR | 6.85 | 14.17 | 2.83 | 3.98 |
|  | FEELNADLFR | 6.33 | 12.65 | 3.00 | 3.85 |
| **P11277** | **Spectrin beta chain, erythrocytic** |  |  |  |  |
|  | RVEDQVNVR | 3.00 | 3.75 | 3.35 | 4.37 |
| **P13716** | **Delta-aminolevulinic acid dehydratase** |  |  |  |  |
|  | GSAADSEESPAIEAIHLLR | 4.57 | 3.62 | 3.57 | 5.13 |
| **P13727** | **Bone marrow proteoglycan** |  |  |  |  |
|  | GNLVSIHNFNINYR | 0.12 | 0.20 | 2.85 | 3.93 |
| **P16152** | **Carbonyl reductase [NADPH] 1** |  |  |  |  |
|  | LFSGDVVLTAR | 4.78 | 14.66 | 2.85 | 4.39 |
| **P16157** | **Ankyrin-1** |  |  |  |  |
|  | AEDSDATGHEWK | 2.86 | 4.82 | 2.75 | 4.34 |
|  | DSGEGDTTSLR | 3.16 | 3.56 | 3.30 | 4.00 |
|  | DVDEEKELLDFVPK | 21.94 | 34.41 | 3.33 | 5.00 |
|  | ELQFSVEDINR | 4.44 | 7.00 | 3.05 | 3.90 |
|  | GNTALHIAALAGQDEVVR | 4.94 | 7.21 | 3.35 | 4.89 |
|  | HGVMVDATTR | 80.16 | 165.43 | 3.19 | 3.86 |
|  | IITTDFPLYFVIM | 4.19 | 9.61 | 2.81 | 4.23 |
|  | ITHSPTVSQVTER | 10.15 | 10.05 | 2.59 | 4.11 |
|  | LEGALSEEPR | 5.37 | 10.11 | 3.44 | 4.16 |
|  | QNQVEVAR | 7.09 | 11.67 | 2.31 | 2.94 |
|  | RQDDATGAGQDSENEVSLVSGHQR | 68.60 | 76.42 | 3.09 | 5.32 |
|  | RTPTPLALR | 20.09 | 16.54 | 2.41 | 3.58 |
|  | SENGSVWK | 3.09 | 3.63 | 2.31 | 3.04 |
|  | VENPNSLLEQSVALLNLWVIR | 3.57 | 6.50 | 4.31 | 6.15 |
| **P16452** | **Erythrocyte membrane protein band 4.2** |  |  |  |  |
|  | NPPENTFLR | 4.34 | 5.17 | 2.24 | 3.22 |
| **P17987** | **T-complex protein 1 subunit alpha** |  |  |  |  |
|  | SQNVMAAASIANIVK | 4.81 | 7.43 | 2.62 | 5.78 |
| **P21980** | **Protein-glutamine gamma-glutamyltransferase 2** | |  |  |  |
|  | NEFGEIQGDK | 4.94 | 6.66 | 2.39 | 3.40 |
| **P27105** | **Erythrocyte band 7 integral membrane protein** |  |  |  |  |
|  | LPVQLQR | 2.91 | 7.41 | 2.29 | 2.81 |
| **P28066** | **Proteasome subunit alpha type-5** |  |  |  |  |
|  | EELEEVIKDI | 23.09 | 16.66 | 2.74 | 3.75 |
|  | LFQVEYAIEAIK | 6.37 | 5.06 | 2.74 | 4.99 |
| **P31939** | **Bifunctional purine biosynthesis protein PURH** |  |  |  |  |
|  | AFTHTAQYDEAISDYFR | 14.59 | 24.33 | 2.91 | 4.46 |
|  | HVSPAGAAVGIPLSEDEAK | 112.03 | 126.52 | 2.63 | 4.61 |
| **P35579** | **Myosin-9** |  |  |  |  |
|  | ALEQQVEEMK | 0.14 | 0.24 | 2.70 | 3.76 |
| **P35998** | **26S protease regulatory subunit 7** |  |  |  |  |
|  | GVLLFGPPGTGK | 17.73 | 13.19 | 2.43 | 3.29 |
| **P43034** | **Platelet-activating factor acetylhydrolase IB subunit alpha** | |  |  |  |
|  | EEFTSGGPLGQK | 5.41 | 11.66 | 2.40 | 3.45 |
| **P45974** | **Ubiquitin carboxyl-terminal hydrolase 5** |  |  |  |  |
|  | EVQDGIAPR | 14.42 | 28.44 | 2.29 | 2.81 |
|  | IVILPDYLEIAR | 8.92 | 12.09 | 2.47 | 3.73 |
| **P48426** | **Phosphatidylinositol 5-phosphate 4-kinase type-2 alpha** | |  |  |  |
|  | DNDFINEGQK | 12.39 | 14.70 | 2.80 | 3.20 |
|  | HGAGAEISTVNPEQYSK | 17.56 | 18.74 | 3.11 | 5.15 |
|  | LMDYSLLVGIHDVER | 16.12 | 16.38 | 2.71 | 3.72 |
| **P48506** | **Glutamate--cysteine ligase catalytic subunit** |  |  |  |  |
|  | VVINVPIFK | 10.16 | 12.66 | 2.43 | 3.32 |
| **P49913** | **Cathelicidin antimicrobial peptide** |  |  |  |  |
|  | FALLGDFFR | 0.04 | 0.09 | 2.48 | 3.27 |
| **P50990** | **T-complex protein 1 subunit theta** |  |  |  |  |
|  | APGFAQMLK | 4.76 | 8.95 | 2.50 | 3.74 |
|  | AVDDGVNTFK | 4.02 | 12.57 | 2.56 | 4.07 |
|  | DIDEVSSLLR | 11.66 | 21.76 | 2.76 | 3.95 |
| **P50991** | **T-complex protein 1 subunit delta** |  |  |  |  |
|  | FSNISAAK | 22.09 | 35.45 | 2.50 | 3.02 |
|  | GIEILTDMSRPVELSDR | 26.44 | 56.97 | 2.70 | 4.46 |
| **P53004** | **Biliverdin reductase A** |  |  |  |  |
|  | NPHPSSAFLNLIGFVSR | 14.99 | 31.93 | 3.07 | 4.58 |
|  | SPLSWIEEK | 6.09 | 16.72 | 2.71 | 3.64 |
| **P55072** | **Transitional endoplasmic reticulum ATPase** |  |  |  |  |
|  | DVDLEFLAK | 6.78 | 12.41 | 2.24 | 3.16 |
|  | ELQELVQYPVEHPDK | 23.22 | 20.14 | 2.73 | 4.29 |
|  | KYEMFAQTLQQSR | 4.94 | 6.86 | 3.07 | 5.26 |
|  | VINQILTEMDGMSTK | 5.74 | 8.28 | 2.44 | 5.95 |
| **P59665** | **Neutrophil defensin 1** |  |  |  |  |
|  | YGTCIYQGR | 0.28 | 0.30 | 3.04 | 3.67 |
| **P62191** | **26S protease regulatory subunit 4** |  |  |  |  |
|  | IKDYLLMEEEFIR | 6.82 | 15.62 | 2.92 | 4.56 |
| **P62333** | **26S protease regulatory subunit 10B** |  |  |  |  |
|  | HGEIDYEAIVK | 7.14 | 21.38 | 3.41 | 4.58 |
| **P62805** | **Histone H4** |  |  |  |  |
|  | VFLENVIR | 0.15 | 0.36 | 2.40 | 3.25 |
| **P78371** | **T-complex protein 1 subunit beta** |  |  |  |  |
|  | ILIANTGMDTDK | 11.86 | 16.29 | 2.50 | 4.34 |
|  | LALVTGGEIASTFDHPELVK | 16.71 | 29.67 | 2.77 | 4.17 |
|  | LTSFIGAIAIGDLVK | 9.44 | 9.15 | 3.47 | 5.67 |
| **Q00013** | **55 kDa erythrocyte membrane protein** |  |  |  |  |
|  | HSSIFDQLDVVSYEEVVR | 22.36 | 41.47 | 2.77 | 5.71 |
| **Q00610** | **Clathrin heavy chain 1** |  |  |  |  |
|  | ALEHFTDLYDIK | 7.35 | 8.34 | 2.93 | 4.72 |
|  | HELIEFR | 7.79 | 13.41 | 2.28 | 2.99 |
|  | KFNALFAQGNYSEAAK | 25.27 | 19.88 | 3.17 | 5.02 |
|  | LLYNNVSNFGR | 20.04 | 19.56 | 3.00 | 4.21 |
| **Q05DK5** | **ADD2 protein (Fragment)** |  |  |  |  |
|  | VNVADEVQR | 18.89 | 37.69 | 2.49 | 3.76 |
| **Q08495** | **Dematin** |  |  |  |  |
|  | HLIEDLIIESSK | 8.51 | 17.98 | 3.05 | 4.49 |
|  | SPGIISQASAPR | 5.14 | 12.94 | 2.60 | 5.24 |
| **Q13200** | **26S proteasome non-ATPase regulatory subunit 2** | |  |  |  |
|  | LVGSQEELASWGHEYVR | 15.46 | 22.15 | 3.07 | 4.43 |
|  | YLYSSEDYIK | 4.56 | 6.29 | 2.43 | 3.67 |
| **Q13228** | **Selenium-binding protein 1** |  |  |  |  |
|  | HEIVQTLSLK | 11.11 | 20.00 | 2.80 | 3.59 |
|  | NEGGTWSVEK | 10.15 | 15.05 | 2.51 | 3.31 |
| **Q14254** | **Flotillin-2** |  |  |  |  |
|  | LLAELPASVHALTGVDLSK | 26.69 | 22.09 | 2.67 | 4.23 |
|  | SAFSEEVNIK | 10.29 | 11.90 | 2.88 | 3.82 |
| **Q16777** | **Histone H2A type 2-C** |  |  |  |  |
|  | VTIAQGGVLPNIQAV | 0.28 | 0.27 | 2.91 | 4.36 |
| **Q1WWM3** | **EPB41 protein (Fragment)** |  |  |  |  |
|  | DVPIVHTETK | 21.36 | 46.28 | 2.56 | 3.20 |
| **Q53HV2** | **Chaperonin containing TCP1, subunit 7 (Eta) variant (Fragment)** | | |  |  |
|  | LPIGDVATQYFADR | 17.54 | 21.48 | 3.61 | 4.53 |
|  | TFSYAGFEMQPK | 8.03 | 16.76 | 3.16 | 3.89 |
| **Q53S54** | **Putative uncharacterized protein CUL3 (Fragment)** | |  |  |  |
|  | NAIQEIQR | 6.21 | 11.55 | 2.34 | 2.88 |
| **Q59ET3** | **Chaperonin containing TCP1, subunit 6A isoform a variant (Fragment)** | | | |  |
|  | ALQFLEEVK | 20.75 | 39.35 | 2.74 | 3.64 |
|  | GFVVINQK | 38.03 | 97.81 | 2.39 | 2.89 |
| **Q5TDH0** | **Protein DDI1 homolog 2** |  |  |  |  |
|  | DMLLANPHELSLLK | 7.55 | 11.89 | 2.79 | 5.22 |
|  | LFSADPFDLEAQAK | 5.21 | 8.05 | 3.55 | 5.22 |
|  | VLVEQQQDR | 2.72 | 4.42 | 2.46 | 3.56 |
| **Q5VVQ6** | **Ubiquitin thioesterase OTU1** |  |  |  |  |
|  | GLTGQAEAR | 2.47 | 7.03 | 2.61 | 3.41 |
| **Q5XPI4** | **E3 ubiquitin-protein ligase RNF123** |  |  |  |  |
|  | LEDANLPSLQK | 12.65 | 35.33 | 3.05 | 4.10 |
| **Q6XQN6** | **Nicotinate phosphoribosyltransferase** |  |  |  |  |
|  | ALAQLSLSR | 10.65 | 14.69 | 2.62 | 3.30 |
|  | SPAQYQVVLSER | 19.38 | 27.03 | 3.13 | 3.94 |
| **Q86UX7** | **Fermitin family homolog 3** |  |  |  |  |
|  | VVLAGGVAPALFR | 11.53 | 14.30 | 2.52 | 4.38 |
| **Q86X55** | **Histone-arginine methyltransferase CARM1** |  |  |  |  |
|  | GAAVDEYFR | 9.71 | 12.93 | 2.71 | 3.37 |
| **Q8WW22** | **DnaJ homolog subfamily A member 4** |  |  |  |  |
|  | DVYDQGGEQAIK | 13.61 | 15.19 | 3.26 | 4.24 |
|  | ITDDMDQVELK | 18.09 | 28.32 | 3.05 | 4.25 |
|  | LISQAYEVLSDPK | 16.38 | 20.13 | 3.12 | 4.93 |
| **Q9BRA2** | **Thioredoxin domain-containing protein 17** |  |  |  |  |
|  | YEEVSVSGFEEFHR | 11.50 | 22.39 | 3.21 | 4.43 |
| **Q9C0C9** | **(E3-independent) E2 ubiquitin-conjugating enzyme** | |  |  |  |
|  | VEVVWADNSK | 7.33 | 12.67 | 2.46 | 3.61 |
| **Q9UBQ7** | **Glyoxylate reductase/hydroxypyruvate reductase** | |  |  |  |
|  | ILDAAGANLK | 12.80 | 27.38 | 2.43 | 3.55 |
| **Q9Y230** | **RuvB-like 2** |  |  |  |  |
|  | AAGVVLEMIR | 7.05 | 12.30 | 2.53 | 3.45 |
|  | LLIVSTTPYSEK | 15.39 | 18.82 | 3.06 | 3.76 |
| **R4GNH3** | **26S protease regulatory subunit 6A** |  |  |  |  |
|  | GVLMYGPPGTGK | 5.21 | 10.33 | 2.43 | 3.40 |
|  | VDILDPALLR | 6.94 | 15.38 | 2.43 | 3.21 |
