# Supplementary material for: Whole Blood Storage in CPDA1 Blood Bags Alters Erythrocyte Membrane Proteome
Source: Oxid Med Cell Longev. 2018 Nov 8;2018:6375379. doi: 10.1155/2018/6375379 (PMC6249999; doi:10.1155/2018/6375379)
Supplement: Supplementary 4 — Table S4: quantitative fold change of proteins commonly identified for Bayesian and Wilcoxon statistical analyses. [file 6375379.f4.docx]

**Table S4.** Quantitative fold-change of proteins commonly identified for Bayesian and Wilcoxon statistical analysis

| Common proteins (Bayes & Wilcoxon)  Increasing quantitatively | Fold change after 14 days | Fold change after 35days |
| --- | --- | --- |
| A0A024RC87 | 2.74 | 3.28 |
| Q5TDH0 | 4.52 | 6.01 |
| A0A024R8I2 | 3.19 | 5.34 |
| Q6XQN6 | 6.80 | 7.88 |
| P04921 | 5.65 | 9.07 |
| Q8IUI8 | 7.91 | 4.28 |
| O95373 | 6.37 | 9.01 |
| Q9H2T7 | 8.03 | 13.70 |

| Common proteins (Bayes & Wilcoxon)  Decreasing quantitatively | Fold change after 14 days | Fold change after 35 days |
| --- | --- | --- |
| Q8IUE6 | 0.20 | 0.30 |
| P02776 | 0.04 | 0.04 |
| P68366 | 0.22 | 0.18 |
| P49913 | 0.08 | 0.01 |
| Q9Y490 | 0.15 | 0.23 |
| A0A140GX60 | 0.06 | 0.06 |
